# Large diversity in the O-chain biosynthetic cluster within populations of Pelagibacterales

**DOI:** 10.1128/mbio.03455-24

**Published:** 2025-02-19

**Authors:** Jose M. Haro-Moreno, Mario López-Pérez, Carmen Molina-Pardines, Francisco Rodriguez-Valera

**Affiliations:** 1 Evolutionary Genomics Group, División de Microbiología, Universidad Miguel Hernández, Alicante, Spain; University of Vienna, Vienna, Austria

**Keywords:** SAR11, Pelagibacterales, O-antigen, lipopolysaccharide (LPS), single-amplified genomes, genomic islands, PacBio metagenomics

## Abstract

Genomic diversity in prokaryotic species is largely due to the existence of extensive pangenomes, allowing different gene complements to be drawn depending on the strain. Here, we have studied the diversity of the O-chain polysaccharide biosynthesis cluster (OBC) in marine bacteria of the Pelagibacterales order as a proxy to measure such genetic diversity in a single population. The study of single-amplified genomes (SAGs) from the whole order found a pattern similar to that of other well-studied microbes, such as the Enterobacteriales or *Alteromonas*, where distinct OBCs represent strains containing different gene pools. We found that most of the OBC sharing happened among individuals of the same clonal frame (>99% average nucleotide identity). Moreover, given the parsimonious way this cluster changes, the diversity of the OBCs can be extrapolated to the size of the population’s pangenome. This assumes that different OBCs correspond to lineages containing unique flexible gene pools, as seen in the aforementioned microbes. Through long-read metagenomics, we could detect 380 different OBCs at a single Mediterranean sampling site. Within a single population (single species and sample) of the endemic Ia.3/VII (gMED) genomospecies, we identified 158 OBCs, of which 130 were unique. These findings suggest that the gene pool within a single population might be substantial (several thousands). While this figure is large, it aligns with the complexity of the dissolved organic matter that these organisms can potentially degrade.

**IMPORTANCE:**

Different strains of the same bacterial species contain very different gene pools. This has been long known by epidemiologists. However, it is unknown what gene pool is present in a single set of environmental conditions, i.e., the same time and place in free-living bacteria. Here, we have leveraged information from SAGs to analyze the diversity of the gene cluster coding for the O-chain polysaccharide, a typical component of the flexible gene pool classically used as a tool to differentiate strains in clinical microbiology. It evolves at a similar rate to the rest of the genome and does not seem to be affected by an arms race with phages. One single species of Pelagibacteriales (gMED) revealed an astounding diversity in one sample studied by long-read metagenomics. Our results point to a large gene pool (local pangenome) present in a single population, which is critical to interpreting the biological meaning of the pangenome, *i.e*., it provides intrapopulation diversity rather than characterizing strains with different distribution in time and/or space.

## INTRODUCTION

Determining the diversity of strains within populations of bacteria has been a major conundrum in microbiology (1). Some recent studies in the gut microbiome by culture (2), metagenomics (3–5), and long-read amplicons of flagellins (6) have concluded that few strains of each species dominate the population within a single individual human microbiome and that they are relatively stable through time. However, studies from metagenomics and single-cell genomics indicated that diversity within a single drop of seawater might be very high (7–9). Nevertheless, even the orders of magnitude of such diversity were hard to establish due to the small size of metagenomic reads or the incompleteness of single-amplified genomes (SAGs). A recent study by culture and metagenomics in an extreme aquatic environment revealed a very high strain diversity in *Salinibacter ruber* with several hundreds of strains within a single pond (1).

In a recent review (10), it was calculated that more than 400,000 independent metabolic pathways were required to metabolize marine dissolved organic matter (DOM), and several hundreds of thousands of ATP-binding cassette (ABC) transporters have been identified in marine metagenome assemblies (11). Thus, it could be expected that a single species of a dominant heterotrophic microbe, such as *Pelagibacter*, could contain a large diversity of genes in its local pangenome, but the diversity of transporters could also be distributed across many different species.

A very common (if not universal) component of the flexible genome (variable from one strain to another within the same species) is the gene cluster coding for the synthesis of external polysaccharides (12, 13), such as the one containing the genes synthesizing the O-chain of the lipopolysaccharide (often referred to as O-antigen) (14–16). The product of this gene cluster is the outermost polysaccharide in the lipopolysaccharide molecules of Gram-negative bacteria cell walls. The high variability of this outer membrane component has been known for long and used in pathogenic/saprophytic bacteria (17–20) to type strains in epidemic outbreaks (serotyping), assuming that each strain has a unique combination of exposed polysaccharides that can be detected with specific antibodies. However, the annotation of the genes involved is not trivial given the complexity of the polysaccharide biosynthesis, involving monomer synthesis and modification, membrane transport and polymerization (15), and their enormous sequence diversity. Fortunately, from a genomic perspective, most of the genes involved in the O-chain biosynthesis form a large gene cluster (OBC) in most Gram-negative bacteria facilitating its identification.

In the order Enterobacteriales, a recent study by Holt and colleagues (21) clearly illustrates the patterns of variation found at these organisms’ major glycotype (combinations of exposed polysaccharides) gene clusters. After analyzing more than 27,000 genomes, they shed light on the evolution of these loci. Their enormous diversity (ca. 18,000 different OBCs were described) and rare exchange between different strains explain their discriminating power in epidemiology to identify outbreaks produced by the same strain. On the other hand, when HGT was detected, the divergence of the genes indicated old transfers (long-term preservation), rare events that do not affect the association between OBC and strain (21). These results also discredit the view that the diversity of OBCs is explained by phage–host arms race. For that, a fast change would be expected, as detected in laboratory experiments in which mutation or inactivation of one gene suffice to generate phage resistance (22, 23).

Here, we have focused on a study of O-chain biosynthetic gene cluster (OBC) diversity in the marine order Pelagibacterales, taking advantage of the abundance of this oligotrophic microbe in SAG and metagenomic databases and the use of long-read (PacBio HiFi) metagenomics. Their photoheterotrophic lifestyle is well known and makes them among the most relevant microbes in nutrient fluxes in the oligotrophic ocean (24–27). Besides, their streamlined genome size of ~1.3 Mb (one of the smallest for planktonic free-living microbes) (26, 28) simplifies the analysis, as they contain a single gene cluster involved in O-chain biosynthesis (e.g., no capsular envelope has ever been found, and they have no flagella that could be glycosylated). Furthermore, the cluster is delimited by the two parts of the ribosomal RNA operon that is split into two sections, 16S–23S genes on one side and 5S gene on the other (27), which provides good markers. In addition, a fine taxonomy derived from the 16S–23S internal transcribed spacer (ITS) can be used to classify the cognate genome with high reliability (29), and the rRNA genes can be used as hallmarks for identifying Pelagibacterales reads in long-read metagenomes (30, 31). This way, we have been able to dissect the diversity of OBC gene clusters as a whole and then also at the population (one single species in one single sample) level. Our findings about the diversity of these OBCs indicate that indeed strain diversity can be very high, even at the single population level (a large local pangenome).

## RESULTS

### OBCs variation across the order Pelagibacterales

Our first objective has been the analysis of the evolutionary dynamics in the available diversity of Pelagibacterales OBCs to check if the pattern described previously for saprophytic microbes (Enterobacteriales) could be extrapolated to free-living ones that never interact with immune systems. We screened a data set comprising nearly 1,700 marine SAGs (32–36) and 20 marine isolate genomes from the whole order Pelagibacterales (clades Ia, Ib, Ic IIa, IIb, and IIIa) (Fig. S1). We avoided clades IV and V, the latter also known as HIMB59, since they have been recently classified as different orders (33, 37, 38). By using the 16S–ITS–23S rRNA operon and the 5S rRNA gene at the left and right ends as hallmarks, we could recover 806 OBCs > 10 kb long (Fig. S1A). The Pelagibacterales are characterized by unusually high synonymous replacements (37), and thus, the 95% average nucleotide similarity (ANI) used for bacterial species definition (39) gives a very split taxonomy that contrasts with their high synteny and coverage values (33). Thus, we have used a genomospecies classification based on phylogenomics and environmental distribution (33).

After manual curation, a major synteny break in the location of the OBC in genomes from the genomospecies Ib.4. In this genomospecies, the OBC was found between the 16S–ITS–23S at one end (like in the other clades), but at the other end, it was located near a cluster of genes involved in the peptidoglycan biosynthesis (Fig. S2A) and two tRNAs coding for valine and methionine. The 5S rRNA gene is found elsewhere (Fig. S2A). This OBC location is ~330 kb distant from the OBC locus in the other clades. A total of 27 SAGs, all in the Ib.4, contained the relocated island. Given that there is no complete genome available for this genomospecies, we collected into a single contig three almost identical (>99% ANI) SAGs into a partially complete (1.06Mb) and admittedly chimeric construct (40, 41). Fig. S2B shows the reconstructed genomic fragment, indicating the position of the 16S, 23S, and 5S rRNA genes. Metagenomic under-recruitment confirmed that the region in Fig. S2A corresponded indeed to the OBC found in other clades that have been translocated to one of the tRNA genes present there. tRNAs are frequent targets of site-directed non-homologous recombination. Still, these OBCs were used for the subsequent diversity comparisons and showed very similar behavior (see below).

Morever, 163 islands (one-fifth of the data set) were categorized as complete, including eight from the rearranged OBCs found in Ib.4. The remaining were partial islands, from which we could recover only the left-hand side (~23%), right-hand side (~22%), or both sides but in different contigs within the same SAG (~35%) (Fig. S1A; Table S1). Regarding their origin, nearly half of the OBCs (#373) were recovered from a single sample in the Bermuda Atlantic Time-series Study (BATS) (sample SWC-09) collected from a 10 m deep water-column sample (Fig. S1B) (34). Therefore, we can consider all these SAGs as belonging to a single community and those within a single species as members of a single population (in the Neodarwinian sense, i.e., they can exchange genes). The remaining OBCs came from a variety of samples from the Atlantic and Pacific Oceans (#170 and #197, respectively) (32, 34), the Mediterranean (33) (#33), and the Red Seas (#15) (35) (Fig. S1B). We classified the Pelagibacterales genomes containing OBCs as members of the subclades Ia and Ib (~90% of the OBCs), and, in much smaller numbers, to subgroups Ic, IIa, IIb, and IIIa (Fig. S1C). This was to be expected since most samples were collected from surface waters, where members of subclades Ia and Ib are largely predominant. Members of subclades Ic and II are mainly found in meso- and bathypelagic waters (25, 36, 42), although some members of the subclade IIa have been detected in surface waters (43).

**Fig 1:**
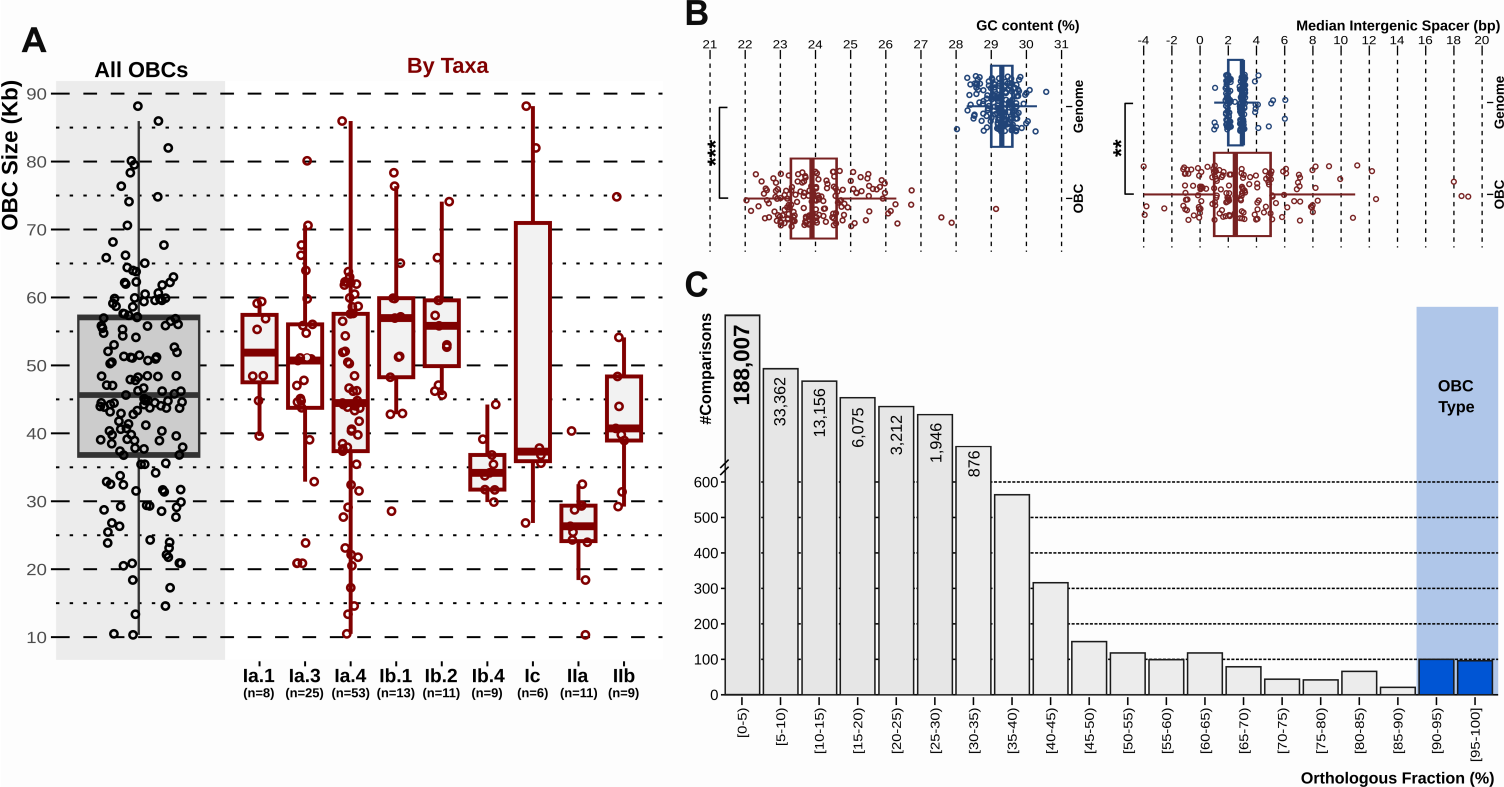
Genomic properties of the 163 complete OBCs and their corresponding genomes. (**A**) Boxplot on the left summarizes the median, first, and third quartiles of the length of the OBC, considering all sequences as a unit (leftmost boxplot) or divided by subgroups. Numbers below the taxonomic name indicate the number of OBCs for each group. Statistics among groups can be found in Table S2. (**B**) Boxplots on the right show the difference in the GC content and the intergenic spacer (upper and lower panels, respectively) between the OBC and its genome. Stars indicate the *P-*value (***, *P* < 0.001; **, *P* < 0.01). (**C**) Histogram resulted from the all-vs-all comparison of the 806 OBCs at 50% amino acid identity threshold. *X*-axis indicates the percentage of orthologous genes (OF) of the shortest sequence (genes shared between two OBCs) in groups of 5% OF.

The analysis of the 163 complete OBCs showed a large size range, from 10 to 90 kb (average size 46 kb) (Fig. 1A; Table S1), which is typical of OBCs regardless of the origin. The number of encoded genes varied from 8 to 101, with an average of 45.7 genes per OBC. Grouped by taxa, the average OBC size varied slightly for members of subclades Ia.1 (53 kb), Ia.3 (51 kb), and Ia.4 (45 kb), while subclades Ib.1 (*n* = 13, 57 kb) and IIa (*n* = 11, 27 kb) had the largest and smallest, respectively (Fig. 1A). Statistically significant differences were detected among subclades (Kruskal–Wallis test, *P* < 0.001), and in more detail from pairs IIa vs Ia.1 (Wilcoxon test, *P* < 0.01), Ia.3 (*P* < 0.05), Ia.4 (*P* < 0.05), Ib.1 (*P* < 0.001), and Ib.2 (*P* < 0.001); and Ib.4 vs Ia.1 (*P* < 0.01), Ib.1 (*P* < 0.05), and Ib.2 (*P* < 0.001) (Table S2). OBCs tend to have a GC content lower than the average of the genome in all Gram-negative bacteria (44). For the Pelagibacterales SAGs described here, regardless of the clade considered, the value for complete OBCs GC content was around 23.8%, statistically significant (paired *t*-test, *P*-value = 1e^−116^), lower than the average GC content of the genomes (29.4%) (Fig. 1B). The median intergenic spacer (very small in streamlined genomes) was also statistically different between genomes and their OBCs (paired *t*-test, *P*-value = 0.003) due to the high dispersion of their values (Fig. 1B). Both differences might reflect the presence of genes of exogenous origin within the OBCs, although it seems difficult to find many microbes with lower GC content than the Pelagibacterales.

### Similarity of shared OBC types

To evaluate the presence of similar OBC ORFs among the Pelagibacterales genomes, we performed an all-vs-all comparison among them (see Materials and Methods) (Table S3). As shown in Fig. 1C, the majority of the comparisons, measured as the fraction of orthologous genes shared among two OBCs at 50% AAI and 70% coverage, showed no similarity. For instance, 75.7% of the OBC pairs shared less than 5% of orthologs, while this number increases to 94.4% if we consider those with less than 15% of orthologous genes (Fig. 1C). These results illustrate the enormous diversity of OBC genes within the Pelagibacterales order, similar to Enterobacteriales, where 18,384 OBCs resulted in only 2,654 (14.4 %) coding for the same gene sets (21). We thus consider two OBCs to belong to the same locus type (hitherto OBC type) similar to reference 21 if they share at least 90% of the genes (Fig. 1C). As a result, from the initial set of 806 OBCs (including incomplete ones), 208 genomes shared the OBC type with another genome in our data set, while 598 genomes had unique (singleton) OBCs. This is typical of the so-called replacement flexible genomic islands that, although coding for a similar function or (in this case) structure, have completely different genetic makeup (13, 45). An advantage of this kind of genomic island for comparative analysis is that incomplete clusters can be used in pairwise comparisons, assuming that similarity throughout some genes can be taken as a sign of a common OBC and likely also a similar (or identical) polysaccharide or glycotype and, at the genome level, expected to have very similar gene pools. This is important when working with SAGs, which tend to be fragmented and incomplete, and even more so for long metagenomic reads (see below).

As expected, either the clusters are syntenic and have >85% similarity, or they are completely different in gene content with nearly no orthologous genes detected (Fig. 1C). For the OBCs belonging to the same OBC type, in most cases, similarity was very high over the whole stretch, but there were exceptions. For example, Fig. S3 shows in one of the pairs, similarity has decreased throughout the right-hand side end of the OBC. In other cases (Fig. S3), variation seems to be concentrated at the left-hand-side end. In these two cases (and in most), variability seems to increase at the ends (one of them), as was the case in the Enterobacteriales (21). This fact improves the reliability of partial sequence comparisons involving only the ends of the OBC, as was done with the metagenomic long reads (see below).

**Fig 2:**
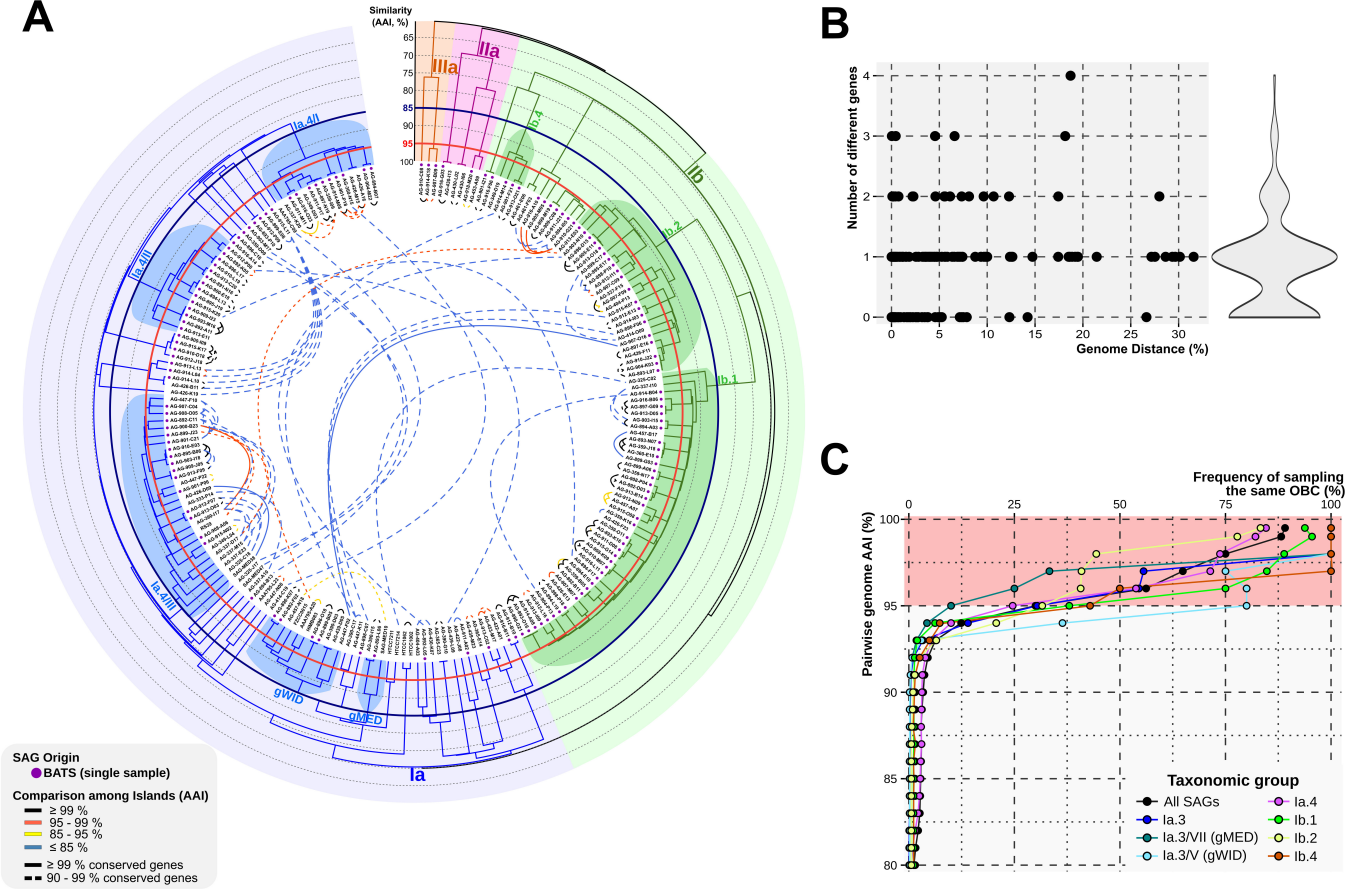
(A) Cladogram-based classification of the 806 genomes that shared OBC type (the complete cladogram is shown in Fig. S4). The outer rings represent the scale of the cladogram, measured as AAI among genomes (hierarchical clustering, average linkage). Red and blue circles mark the Pelagibacterales limits for species suggested in reference 39 (85% ANI) and the more common standard 95%, respectively (at these levels of similarity AAI and ANI are nearly equal). The most relevant species names are used from reference 33 and labeled at the corresponding branching line. Inner connections (>90% shared genes, dashed line; >99%, continuous line) indicate shared OBC types between genomes. Connector colors represent the average identity values of the OBC-shared genes. The major Pelagibacterales subclades are shaded: Ia, blue; Ib, green; II, purple; and IIIa, yellow. A purple dot near the genome name indicates that it comes from a BATS single sample. (**B**) Variation of the number of different genes for the same OBC type between pairs of genomes, expressed as the genomic distance (100, AAI, %). (**C**) Frequency of sampling the same OBC type as a function of the phylogenetic distance between a pair of genomes.

Figure S4 shows an average amino acid identity (AAI) cladogram tree (39) rather than the more common average nucleotide identity (ANI). AAI relationships are helpful when comparing highly divergent genome sequences (46), as those analyzed here for the whole Pelagibacterales order. Figure 2A only shows the genomes sharing OBC types. An expected observation was that, in most cases, SAGs sharing the OBCs were also >99% AAI overall, i.e., they belonged to the same clonal frame or genomovar (1). This result was not surprising, since this is the fundament of strain serotyping of Gram-negative bacteria using O-antigens ( 15, 47). However, a significant number of associations (~13%) were found between individuals belonging to different genomospecies (AAI ~ 85%) from the same family or even from different families (Fig. 2A). There seems to be a bias for certain groups regarding the number of common OBC types. For instance, genomospecies Ia.3/V, also named gWID given that it has a widespread oceanic distribution by metagenomic fragment recruitment (33, 48), is one of the genomospecies with the highest number of retrieved genomes (#79, Fig. S4), but their SAGs barely showed any shared OBC type (#7, Fig. 2A), six of them all corresponded to the same genomospecies. Whereas Ib.1, with 87 genomes, had 37 sharing events, five of them shared between genomes with <95% AAI and a single OBC jumping to another genomospecies. However, most OBC type sharing was detected only among members of the same genomospecies and often between genomes with >95% AAI (Fig. 2A). Only 49 OBC types were found across genomes with less than 95% AAI (31 of the OBC pairs having more than 95% AAI, despite the difference across the rest of the genome). Most shared OBC types had the same gene content or only varied in one gene, regardless of the dissimilarity between genomes sharing the OBC (Fig. 2B). Finally, the rate of shared OBC types (Fig. 2C) drops dramatically below 95% AAI at the genome level, regardless of the taxa, and was negligible (24 OBCs) in genomes with less than 90% AAI. The same pattern applies even when considering individual taxa, i.e., members of the same genomospecies with less than 90% AAI very seldom share OBC type (Fig. 2C).

**Fig 3:**
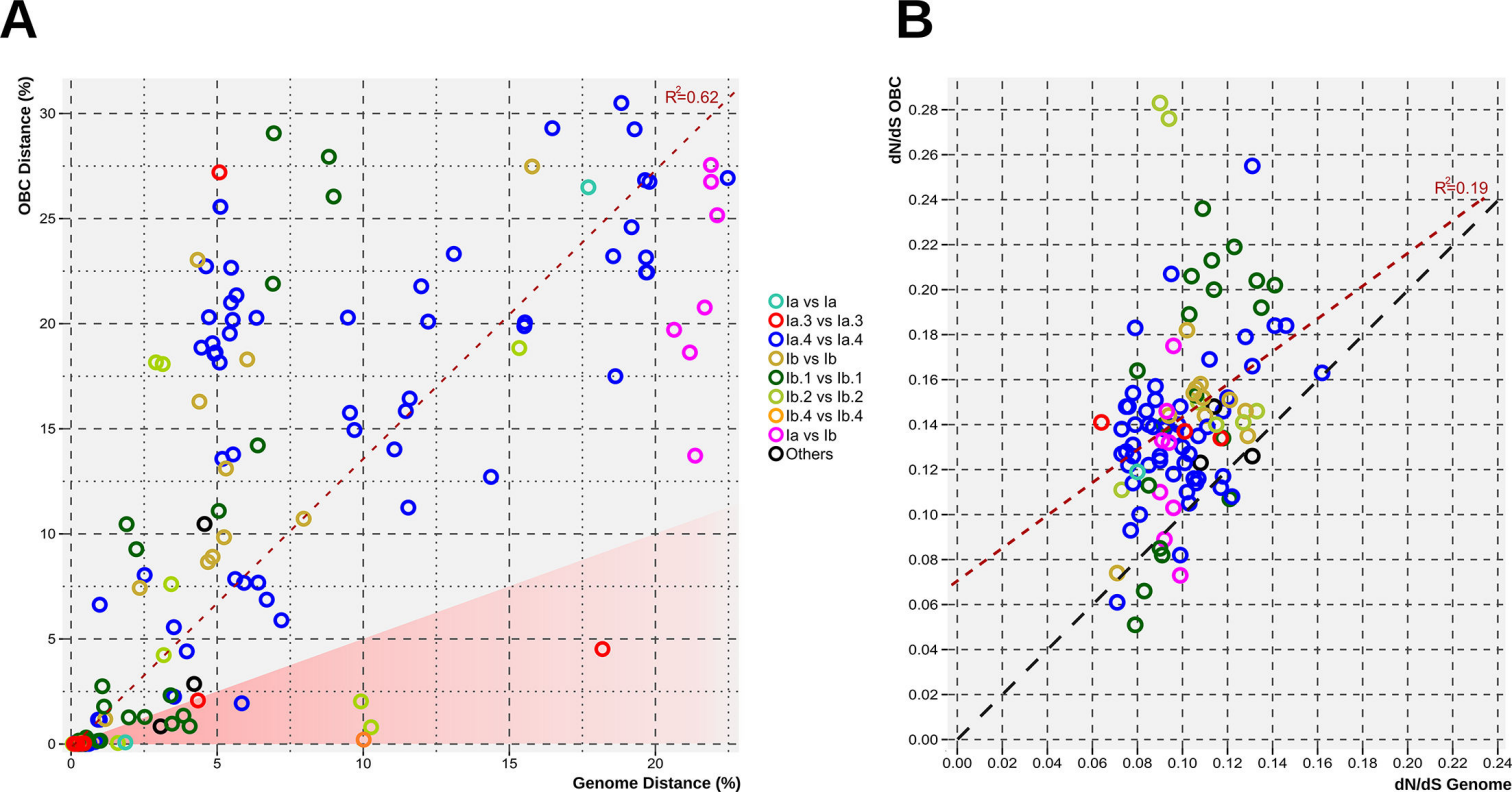
(A) Scatterplot of the relationship between genome distance and locus distance, both expressed as 100 – AAI (%), for only those genomes sharing an OBC type. The dashed line in the scatterplot represents the linear regression line. The red-shaded area indicates recent horizontal gene transfer events (genome distance is at least twice the OBC distance). Dots colored by taxonomy. (**B**) Scatterplot of the relationship between the dN/dS values for OBCs and genomes. Red dashed line represents the regression line, whereas the black dashed line indicates *y* = *x*.

The finding of O-antigen loci shared among distant families suggests horizontal gene transfer (HGT). However, as seen in Fig. 3A, for most shared OBCs (82%), the locus genetic distance expressed as ANI (data not shown) or AAI was similar to or slightly higher than the genome distance. The dN/dS was statistically higher for shared OBC genes (average 0.14 versus 0.1 for the whole genome, paired t-test, *P* < 1e^−15^) indicating a slightly higher rate of positive selection (Fig. 3B). In any case, the values detected for OBC genes indicate that, if they have been exchanged by HGT, it must have been an old event, and for most of the (evolutionary) time, they are vertically inherited (long-term preservation) as described for the Enterobacteriales (21). The O-chain is a primary target for phages and might be subject to arms race rapid evolution, i.e., change much more rapidly than the rest of the genome to evade phage predation (20, 49). However, the data indicate that this is not achieved by swapping the whole OBC or is very rare, as only 14 (~7% of the total shared OBCs) had an OBC distance that was at least half of the genomic distance, indicative of recent events (Fig. 3A). These results reveal that rather than rapidly exchanging their OBCs as some scenarios suggest (20, 50), most are vertically transmitted over long evolutionary periods. Arms race is more likely to act at the level of mutation or recombination affecting single genes (23).

**Fig 4:**
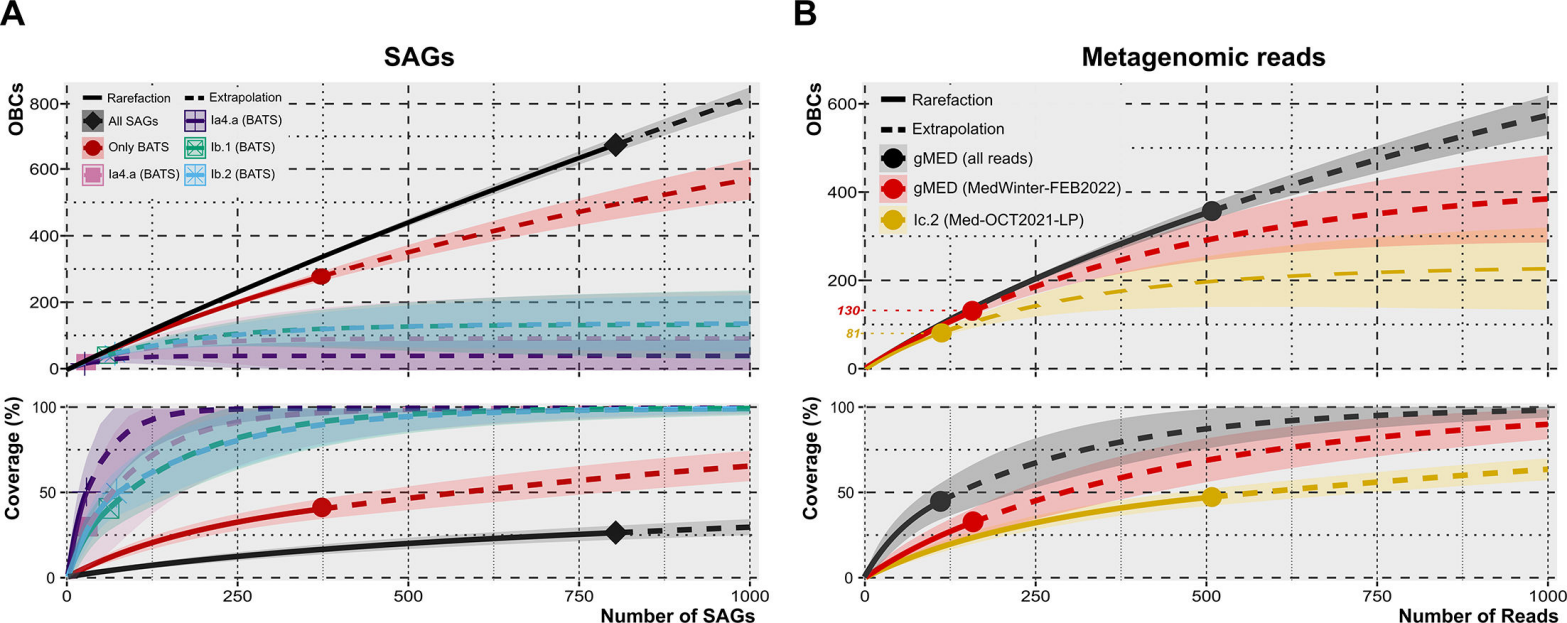
Rarefaction (solid line) and extrapolation (dashed line) curves based on OBC diversity against the number of sequences from (**A**) all SAGs (black), and SAGs coming from the single BATS sample, considering all sequences together (red line) or only the four most representative genomospecies (remaining colored lines); (**B**) PacBio Sequel II metagenomic reads from a set of Mediterranean samples for two abundant genomospecies, gMED in MedWinter-FEB2022 (purple line), and Ic.2 in Med-OCT2021-75m (yellow line). The bottom plots represent how well the number of detected and predicted sequences covered the diversity of OBCs. The dispersion area shows a 95% confidence interval.

Given that nearly half of the SAGs in the database came from a single sample of water in the Sargasso Sea (34), we studied how many different OBC types could be detected at a single location and sample. To do that, we rarefied (51, 52) the genomes and the number of different OBC types detected globally and at BATS. The 373 BATS’ SAGs for the whole Pelagibacterales order had a weak sign of saturation (Fig. 4A). As expected this coverage was even lower (~25%), considering all the 806 genomes for Pelagibacterales as a whole. Therefore, we are far from finding all possible variations in the Pelagibacterales OBCs either in the whole ocean or in a single sample. At the genomospecies level and in the single BATS sample, the numbers were too small to allow for a sensible estimation of the total numbers of OBC types in a single population.

### Diversity of OBCs within a single population by long-read metagenomics

Metagenomic samples sequenced with PacBio Sequel II can be a good alternative to SAGs to study intrapopulation diversity. Given the large size of the reads, typically between 5 and 15 kb long (30), the flexible part of a genome, including fragments presenting part of the OBC, can be retrieved before assembly. As discussed before, retrieving only a few genes allows inferring the OBC type represented by the PacBio read. Thus, we have sequenced and analyzed five different PacBio CCS metagenomes collected from a single location at an off-shore Western Mediterranean Sea off Alicante (see Materials and Methods). We took samples during winter when the water column is fully mixed (MedWinter-JAN2019 [30], MedWinter-FEB2022) and during the stratification season at three different layers in the photic zone, the upper photic (Med-OCT2021-15m), deep chlorophyll maximum (Med-SEP2022-60m, DCM), and lower photic (Med-OCT2021-75m, below DCM). The prokaryotic community at this location has been thoroughly studied by short and long-read metagenomics before (30, 53–55) and the three depths selected when the water column is stratified represent different epipelagic assemblages, as the prokaryotic community significantly differed at these depths (54). Then, we selected all the reads whose 23S rRNA affiliated within the order Pelagibacterales, and hence the left-hand end of the OBCs could be analyzed to study the variability of the OBC types within these samples. Only PacBio CCS reads with at least 5 kb of OBC were used, resulting in a total of 2,780 OBCs from the five metagenomic samples, and the threshold of at least 90% of orthologous genes was applied for their classification into the same OBC type. Thus, we found 2,440 OBC types in all the samples combined (Fig. S5). The resulting rarefaction, extrapolating to 10,000 reads, showed between 4,500 and 6,500 OBC types for the whole Pelagibacterales order at this single sampling site and over the course of a couple of years with the corresponding seasonal variations (Fig. S5). Although numbers are hard to compare, it is certainly in the order of those found for the Enterobacteriales (15,730 in 27,000 genomes) (21).

To be able to classify the recovered OBCs into genomospecies, we have used the internal transcribed spacer (ITS) between the 16S rRNA and 23S rRNA genes. Ecotypes of Pelagibacterales and *Prochlorococcus* have been reliably classified based on their sequence for a long time (29, 56–59). More recently, a clear association between genomospecies classified by phylogenomics and the ITS cladogram was confirmed (33). By examining the correspondence between ITS and 23S rRNA gene sequences, we also determined genomospecies from the latter. Therefore, the long reads with complete ITS (*n* = 1,501) and 23S rRNA sequences (*n* = 2,780) were used to analyze the OBC diversity in single genomospecies (Table 1). As expected, the diversity found within the Pelagibacterales CCS reads was astounding, with many sequences grouping with the main phylogenetic groups (Ia, Ib, Ic, and IIa). In the case of the most abundant genomospecies (by number of OBC reads), Ia.3/VII and Ic.2, their OBC-type numbers were detected at significant values (>5%). Specifically, genomospecies Ia.3/VII had 18.4% of the recovered OBC reads. This group has been characterized as dominating (highest metagenomic recruitment) in epipelagic Mediterranean waters, and hence, it was named gMED (33). By using only the sequences from a single sample (mixed water column, MedWinter-FEB2022) that had the largest number of OBC reads (158 gMED-affiliated sequences), 130 different OBCs were retrieved in this single species population, and the rarefaction curve suggested the presence of around 400 different OBC types (294–480, 95% CI) (Fig. 4B). A similar approach was also applied to another genomospecies, Ic.2 (deep epipelagic), which was found to be abundant (112 out of 474 OBC identified reads) in the lower photic (75 m deep) sample. The rarefaction curve extrapolates to 254 different OBCs (140–310, 95% CI). Although the numbers are smaller than the ones found for the species *Escherichia coli* as a whole (#800 in reference 21), the *E. coli* OBCs were collected from several different populations (e.g., hosts or samples), and the genomic sampling was much larger (ca. 10,000 complete genomes).

**TABLE 1:** Taxonomic classification of PacBio CCS reads containing a 23S rRNA gene (hence an OBC) and classified to the order Pelagibacterales^*b*^

Subclade	Total OBCs	495	458	642	533	652
	Genomospecies	Med-OCT2021-15m	Med-OCT2021-75m	Med-SEP2022-60m	MedWinter-JAN2019	MedWinter-FEB2022
**Ia**	Ia.1/I	5	4	14	5	4
	**Ia.3/VII (gMED)**	**127**	**32**	**65**	**134**	**153^*a*^**
	Ia.3/VII (gWID)	22	9	8	11	17
	Ia.3/Unclassifed	70	38	168	132	115
	Ia.4/I	1	4	1	0	1
	Ia.4/II	0	0	0	1	0
	Ia.4/III	17	5	16	16	16
	Ia.4/Unclassified	79	45	93	79	95
	Ia.Unclassified	14	80	45	16	20
**Ib**	Ib.1	5	1	2	1	2
	Ib.2	17	6	16	17	27
	Ib.3	0	0	0	0	0
	Ib.4	7	3	10	12	16
	Ib.5	22	8	23	9	12
	Ib.Unclassified	10	44	19	15	17
**Ic**	Ic.1	0	0	0	0	0
	**Ic.2**	**5**	**112^*a*^**	**29**	**16**	**10**
**IIa**	—^*c*^	90	61	127	66	140
**IIb**	—	0	5	3	2	4
**IIIa**	IIIa.1	0	0	2	0	3

^*a*^ CCS reads used for rarefaction and extrapolation curves.

^*b*^ Bold text refers to the two genomospecies used for the analysis in Fig. 4B.

^*c*^ ”—” indicates no genomospecies determined for those groups (IIa and IIb).

## DISCUSSION

Polysaccharides are extremely versatile macromolecules in which an infinity of structures and properties can be obtained by small modifications in their sugars, bonding, branching, and length (20). In this sense, they are like proteins, but they have to be synthesized by laborious enzymatic steps exported to the outside of the cell and then polymerized. We have not analyzed the functionality of the genes detected, among other reasons, because they lack in most cases reliable annotation beyond the general description of function (15). However, the whole subject of polysaccharide biosynthesis is challenging even in model organisms (60). Their enormous diversity in the sequence space contributes to the problem. Structural predictions and comparisons could help to solve the conundrum but to produce enough biomass for chemical analysis in hard-to-cultivate microbes, such as *Pelagibacter,* makes it hard and beyond the scope of this work. Thus, we have focused in their gene diversity to assess intraspecies strain diversity rather than the specific mechanisms of cell-wall biosynthesis.

The overall diversity and evolution of OBCs described here in a free-living marine proteobacterial order are very similar to that described using cultures of a heterogeneous group of saprophytic and free-living bacteria like the Enterobacteriales. This proves beyond doubt that the classical view (61, 62) that O-chain diversity is due to the need of the microbes to change their antigenic specificity as protection from host immune systems is misleading, even if this is partially true under some special circumstances. Furthermore, the term “serotype” should be replaced by “glycotype,” which is more realistic (63). The variation at the level of exposed polysaccharides in microbes belonging to the same population has been revealed by metagenomics, culture, and other approaches as a constant feature, at least, for aquatic microbes (13, 41, 64), including Gram positives (40, 65) and even Archaea (66).

The reasons for such extreme flexibility in structures often critical to the survival of cells in nature are still not clear. In free-living prokaryotes, the most obvious reason to have a high diversity of these markers, within a single population, is avoiding predation by protists or phages (67). Although some O-chains have been considered more refractory to protist predation than others (68), there is little doubt that for small cells like those of the Pelagibacterales, the predatory pressure of phages is more important. Something along these lines has been described for the most abundant picocyanobacterium *Prochlorococcus* and its phages (7, 22, 23). The O-chain is a major target for phage receptor-binding proteins and thus there are two potential phage-related explanations for its diversity-arms race and density-dependent negative selection (69, 70). In the latter, increased predation on abundant OBC types maintains a large diversity of receptors, distributing the predation pressure among many different cells. As mentioned before, the swapping of O-chains is too slow to be effective in an arms race scenario. However, at a shorter timeframe, mutation or individual gene or cassette swapping could have a role in the phage–host interaction as seen in several laboratory experiments (23).

What is the relevance of arms race processes then? In the Enterobacteriales, the gain or loss of a small number of genes (1–3) has been described within relatively short (epidemiological) timescales (21). Experimental studies have shown that isolated SNPs can make a strain resistant to one phage and also very small variations in the phage receptor-binding protein can revert the resistance (23, 71). However, it has been shown also by experimental work in cyanophages that a decrease in sensitivity to one phage can increase the sensitivity to another (23). Furthermore, significant changes in the O-chain structure or composition in a Gram-negative bacterium can alter their antibiotic sensitivity (72) and also, likely, its nutritional preferences. O-chain mutations are notoriously pleiotropic, as they have multiple phenotypic effects (73, 74). In addition, the synthesis of an external polysaccharide requires many steps that are connected (synthesis of sugars, transport and linking to the growing external polymer). All these steps must be coordinated and thus submitted to a complexity limitation to change (75). Finally, we would like to suggest that once an equilibrium is reached, any change could lead to a major disruption of the species’ adaptation to its niche (70). In the evolutionary landscape analogy (76), it would imply falling from the mountain peak to the valley and is likely to be very infrequent.

The study of intrapopulation diversity in bacteria is crucial for various fields, including antibiotic resistance, vaccine development, biotechnology, and the food industry (77–79). The role of the prokaryotic species pangenome in bacterial and archaeal biology remains uncertain, partly due to the challenges in defining microbial habitats—particularly in saprophytic Enterobacteriales, which exhibit complex life cycles, broad host ranges, and intricate microniches (80–83). In contrast, Pelagibacterales serve as an ideal model of microbes with a straightforward lifestyle; they are pelagic, non-motile aquatic oligotrophs in the water column (84–86). This study represents an initial step in tackling this challenging yet essential area of research. We have studied the diversity of OBCs within the Pelagibacterales order to discriminate different strains as has been done classically for epidemiology (17–20). We can infer (6) that the number of different strains (cells carrying different gene pools) should be slightly less than the number of different OBCs, i.e., in the order of hundreds of strains within a single population. This number fits well with those found for *Prochlorococcus* by Kashtan and collaborators (8) by comparing SAGs, and it is again surprisingly high for individuals (cells) belonging to the same species and inhabiting such homogeneous environment. They would certainly allow for dealing with large numbers of substrates and conditions by one single species. It has been estimated that dissolved organic matter (DOM) in the ocean contains in the order of 100,000 different chemical formulas, half of which have a half-life time of less than 2 weeks (87). Therefore, the number of genes (e.g., transporters, degradative enzymes) required to cope with such chemical diversity is expected to be very large (10). Thus, it is not surprising that major consumers of DOM, such as Pelagibacterales species, have proportionally large local gene pools or pangenomes. In any case, the use of long-read metagenomics facilitates the recovery of the flexible genome in natural populations, creating potential for untangling the biological role of the prokaryotic pangenome.

## MATERIALS AND METHODS

### **Recovery of Pelagibacterales** genomes

To evaluate the presence and the number of glycosylation islands from the whole Pelagibacterales order (NCBI taxonomy ID 54526), a compendium of nearly 4,100 genome assemblies was downloaded from the NCBI database. Prior to the analysis, due to the incomplete nature of MAGs, only assemblies from SAGs and isolates were considered, and their degree of completeness and contamination of SAGs were estimated using CheckM v1.1.2 (88). SAGs with ≥50% completeness and ≤5% contamination were kept. A fast taxonomic classification of genomes was performed using the GTDB-Tk v2.1.0 tool (89) using the Genome Taxonomy Database (GTDB) release R207 (90). Genomes belonging to clades IV and V (HIMB59) or misclassified were removed from the data set.

### **Genome annotation and retrieval of the O-chain biosynthetic gene cluster** (OBC)

Prodigal v2.6.3 (91) was used to predict genes from contigs retrieved from the individual genomes in the curated Pelagibacterales data set containing 1,700 genomes. Predicted protein-encoded genes were taxonomically and functionally annotated against the NCBI NR database using DIAMOND 0.9.15 (92) and against COG (93) and TIGRFAM (94) using HMMscan v3.3 (95). tRNA and rRNA genes were predicted using tRNAscan-SE v2.0.5 (96) and barrnap v0.92 (https://github.com/tseemann/barrnap), respectively.

Contigs containing a signal for the 23S and 5S rRNA genes were selected as they are characterized as markers for the start and ending points of the OBC in Pelagibacterales. If both markers were found within the same contig, the island was classified as complete. In the end, a total of 806 genomes contained an OBC; 163 were categorized as complete, while the remaining 643 OBCs were partially detected (missing left or right sides, or fragmented). Statistical differences of GC content and intergenic spacers between complete OBCs and their corresponding genomes were calculated with the paired *t*-test function (t.test) from the package stats v4.4.1 in R v4.3.2 (97). In addition, the Kruskal–Wallis test (function kruskal.test) followed with a pairwise comparison using the Wilcoxon test (function pairwise.wilcox.test) were applied to calculate the statistical differences between the sizes of the complete OBCs according to their taxonomic affiliation using the package stats v4.4.1 in R v4.3.2 (97).

### Phylogenomic classification

Using Phylophlan v3.0.67 (98), a total of 104 genes (26,134 amino acid positions) were used to classify the 806 Pelagibacterales genomes containing a complete or partial OBC phylogenomically. Genomes of the HIMB59 order and *Rickettsia* spp. were used as an outgroup. The resulting tree was visualized using iTOL v6 (99). Following the well-established SAR11 nomenclature within subclades, phylotypes, and genomospecies described in references 33 and 100, we used the median distance between nodes and cophenetic correlation coefficient (interval comprised between 0 and 2) to define them.

### Genomic pairwise comparison

Average nucleotide and amino acid identities (ANI and AAI) between a pair of genomes were calculated using the JSpecies v1.2.1 with default parameters (101) and CompareM v0.1.2 (https://github.com/donovan-h-parks/CompareM) software packages respectively. A cladogram of the genomes containing the OBC (complete or partial) was constructed using an all-vs-all matrix of the AAI values among genomes, followed by hierarchical clustering with the average Euclidean distance among values with the hclust function from package stats v4.4.1 in R v4.3.2 (97).

### Determination of O-chain biosynthesis locus types

AAI values between pairs of OBCs were calculated with CompareM, considering 50 % amino acid identity as the threshold to establish similarity among genes. We only considered the pairwise combinations of A (complete OBC) vs A, A vs B (partial OBC), B vs A, A vs C (only left-hand side OBC), C vs A, A vs D (only right-hand side OBC), D vs A, B vs C, C vs B, B vs D, D vs B, C vs C, and D vs D. Two or more OBCs belonged to the same OBC type if they shared at least 90 % of the genes (considering the smallest OBC).

### dN/dS values from OBCs and genomes

Estimation of the numbers of synonymous (dS), non-synonymous (dN) mutations, and the dN/dS ratio was performed using orthologr v0.4.2 (102) against the set of genomes sharing an OBC and their OBCs. The OBC cluster was extracted from the genome sequences before calculation. Ortholog genes were detected using a reciprocal best-hit approach using DIAMOND, and the dN/dS ratios were estimated using the Comeron algorithm (103).

### PacBio CCS15 metagenomic reads

Four marine samples were collected from the same sampling site in the epipelagic Mediterranean Sea at 20 nautical miles off the coast of Alicante (Spain) (37.35361°N, 0.286194°W). MedWinter-FEB2022 (20 m deep) (SRR28395892) was collected during winter, when the water column is fully mixed. Med-OCT2021-15m (SRR28395891), Med-SEP2022-60m (SRR28395890), and Med-OCT2021-75m (SRR28395889) were collected in summer during a strong stratification period. We added to the comparison a winter sample collected in January 2019 and sequenced with PacBio Sequel II (MedWinter-JAN2019 [28] [SRR13009787]). For each depth, 200 L was collected and filtered on board as described in the study of Haro-Moreno et al. (54). Briefly, seawater samples were sequentially filtered through 20, 5, and 0.22 µm pore filter polycarbonate filters (Millipore). Water was directly pumped onto the series of filters to minimize the bottle effect. Filters were immediately frozen on dry ice and stored at −80°C until processing.

DNA extraction was performed from the 0.22 µm filter (free-living bacteria) following the MagAttract Purification Kit protocol (QUIAGEN). Metagenomes were sequenced using PacBio Sequel II (one 8M SMRT Cell Run, 30 h movie) (Novogene, South Korea). To improve the quality of the PacBio raw reads, we generated highly accurate single-molecule consensus reads (CCS reads) using the CCS v6 program of the SMRT-link package. The minimum number of full-length subreads required to generate a CCS read was set to 15 (>99.99% base call accuracy).

### ITS and 23S phylogenies of Pelagibacterales genomes and PacBio CCS reads

PacBio CCS15 reads >5 kb were screened to detect 16S and 23S rRNA genes using barrnap. Using SILVA R138 (104), sequences affiliated with Pelagibacterales were kept, and complete internal transcribed spacers (ITS) and 23S rRNA genes were extracted for further analysis. Phylotype classifications based on the ITS and 23S rRNA gene were inferred using the neighbor-joining approach in MEGA11 (105), with 1,000 bootstraps and the Jukes–Cantor model of substitution. Phylotype assignment followed existing ITS and 23S nomenclatures (24, 29, 57).

### Rarefaction curves among O-antigen loci

We quantified the number of locus types, aka locus diversity, by applying an ecological modeling approach using the iNEXT v3.0.0 package in R (52). In this approach, each SAG or CCS read was considered ecological “sites,” and the OBC types were considered the observed “species” in those sites. The rarefaction curves were calculated by extrapolating our data to 1,000 SAGs and CCS reads for measuring at the genomospecies level and to 10,000 CCS reads for the whole Pelagibacterales order.

## Data Availability

Metagenomic datasets have been submitted to NCBI SRA and are available under BioProject accession number PRJNA1088973 (PacBio CCS15 reads: MedWinter-FEB2022-CCS [SAMN40517308], Med-OCT2021-15m-CCS [SAMN40517305], Med-SEP2022-60m-CCS [SAMN40517307] and Med-OCT2021-75m-CCS [SAMN40517306]).

## SUPPLEMENTAL MATERIAL

Supplemental Figures mbio.03455-24-s0001.pdf Figures S1 to S5.

Table S1 mbio.03455-24-s0002.xlsx Summary of Pelagibacterales genomes with OBCs.

Table S2 mbio.03455-24-s0003.xlsx Summary of statistical tests.

Table S3 mbio.03455-24-s0004.xlsx Summary of pairwise comparisons among OBCs.

ASM does not own the copyrights to Supplemental Material that may be linked to, or accessed through, an article. The authors have granted ASM a non-exclusive, world-wide license to publish the Supplemental Material files. Please contact the corresponding author directly for reuse.
