# Supplementary material for: Large diversity in the O-chain biosynthetic cluster within populations of Pelagibacterales
Source: mBio. 2025 Feb 19;16(3):e03455-24. doi: 10.1128/mbio.03455-24 (PMC11898729; doi:10.1128/mbio.03455-24)
Supplement: Supplemental Figures — Figures S1 to S5. [file mbio.03455-24-s0001.pdf]

Supporting Information for

Large diversity in the O-chain biosynthetic cluster within populations of Pelagibacterales

Jose M. Haro-Moreno[1], Mario López-Pérez[1], Carmen Molina-Pardines[1], and Francisco Rodriguez-Valera[1,*]

[1]Evolutionary Genomics Group, División de Microbiología, Universidad Miguel Hernández, Apartado 18, San Juan 03550, Alicante, Spain.

*Corresponding author: frvalera@umh.es

Universidad Miguel Hernández, División de Microbiología, Apartado 18, San Juan de Alicante, 03550 Alicante, Spain.

Phone +34-965919313, Fax +34-965 919457

This PDF file includes:

Figures S1 to S5.

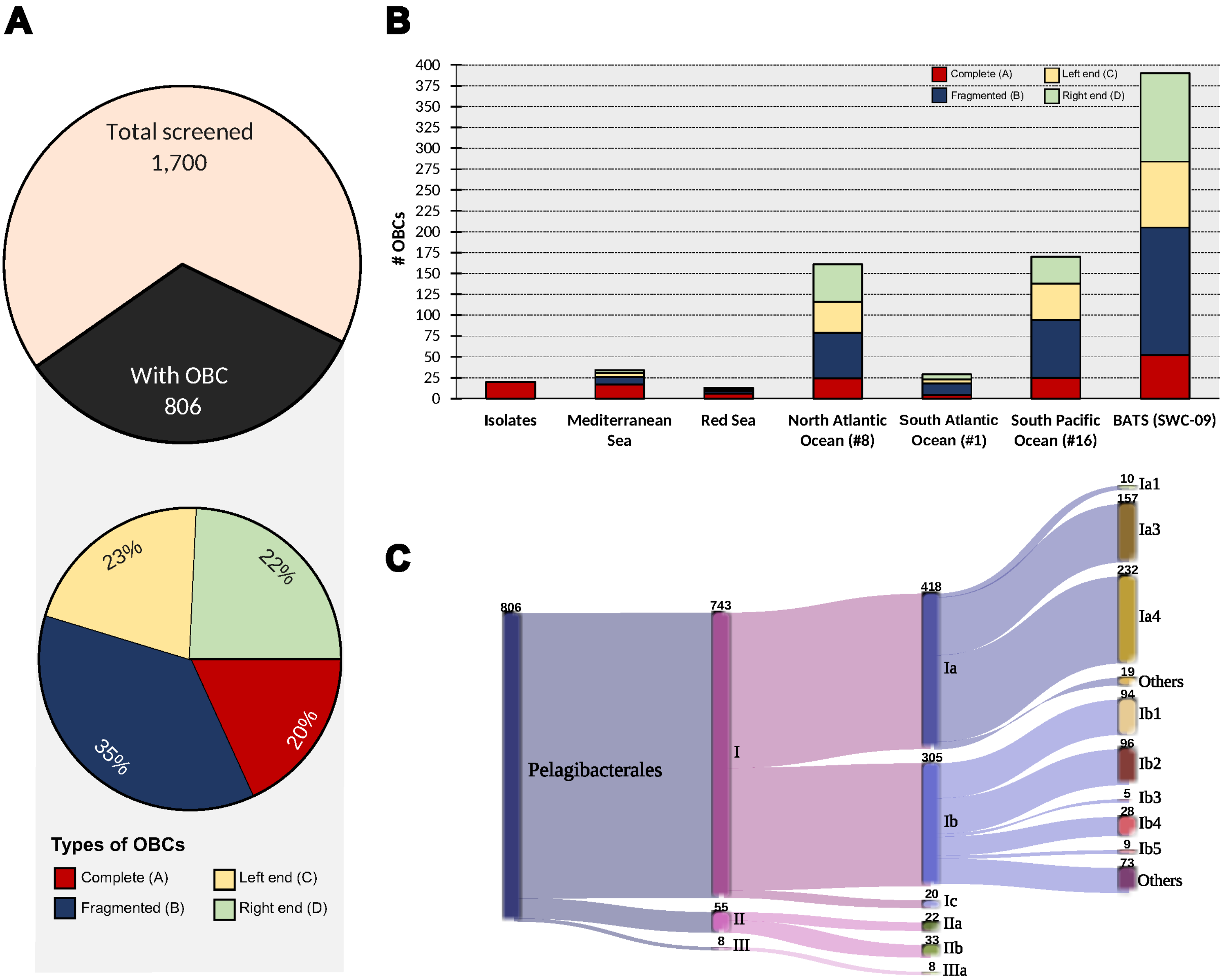

Figure S1. A. Upper pie chart indicates the total number of Pelagibacterales genomes screened (pale color), and the number of genomes on which we could identify the O-chain biosynthetic gene cluster (OBC) (black area). The bottom pie chart distributes the 806 OBCs according to their completeness: A – complete OBC; B – the boundaries of the OBC, i.e. the 23S rRNA gene on the left-hand side and the 5S rRNA gene on the right-hand side, were detected, but in two different contigs from the same genome; C – only the left-hand side; D – only the right-hand side. **B.** Number of OBCs recovered by oceanic region. **C.** Taxonomic classification of Pelagibacterales-containing OBCs based on a maximum-likelihood phylogenetic tree from shared proteins (see methods). The resulting phylogenetic groups follow the nomenclature described in Haro-Moreno et. al., 2020 (32).

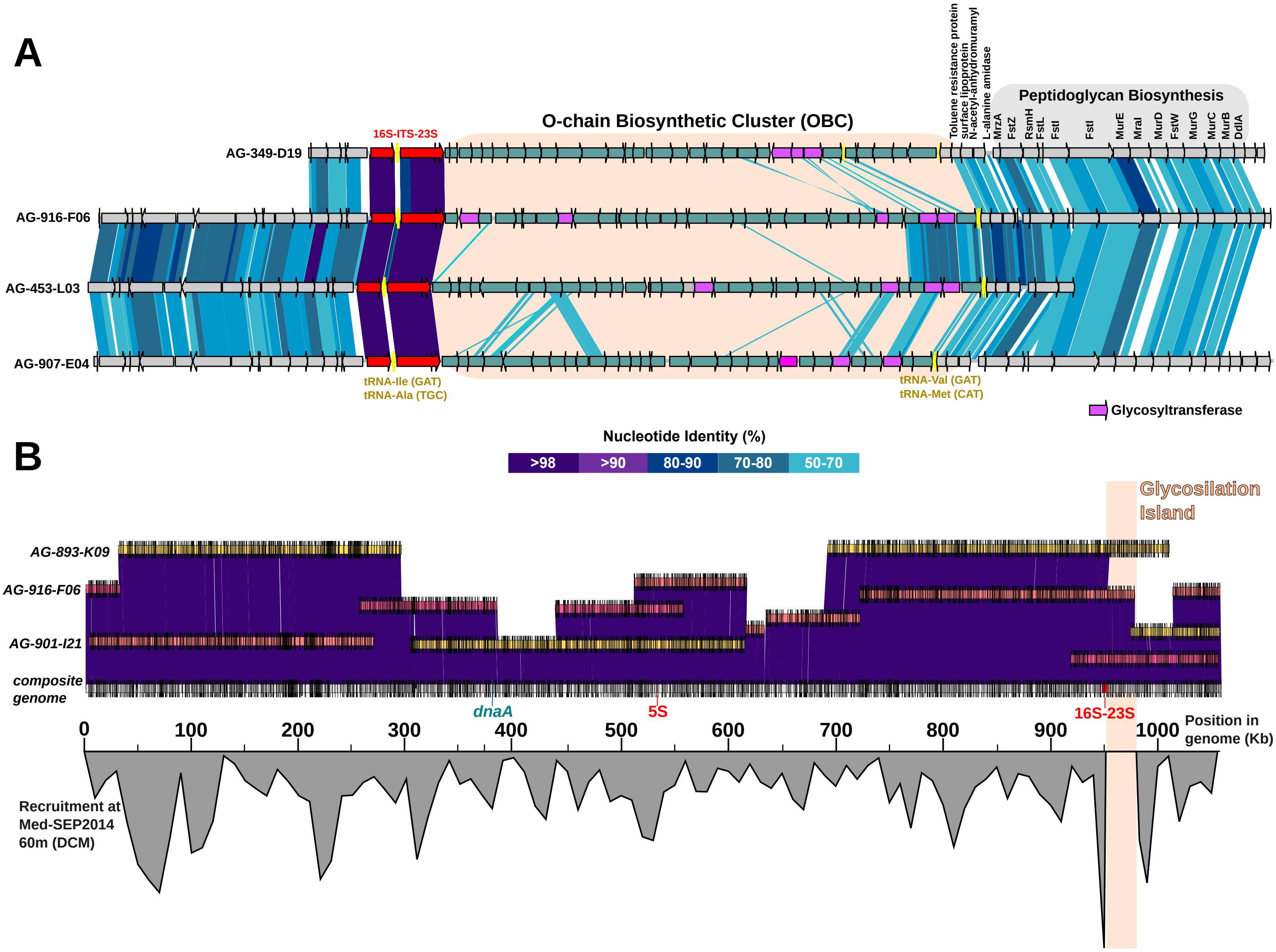

Figure S2. A. Genomic comparison of four selected complete OBCs from members of the Ib.4 phylogroup. Note that in this case, the glycosylation island is located between the 16S-ITS-23S rRNA genes and the tRNA-Val, tRNA-Met genes, and the core genes involved in the peptidoglycan biosynthesis. **B.** Reconstruction of a partial genome, Ib4-rB, in a single contig after the co-assembly of 3 nearly identical (>99 % ANI) SAGs. The locations of the dnaA, 16S, 23S, and 5S rRNA genes are indicated. The metagenomic fragment recruitment of this genome in the Mediterranean Sea (Med-SEP2014-60m) confirmed the location of the glycosilation island.

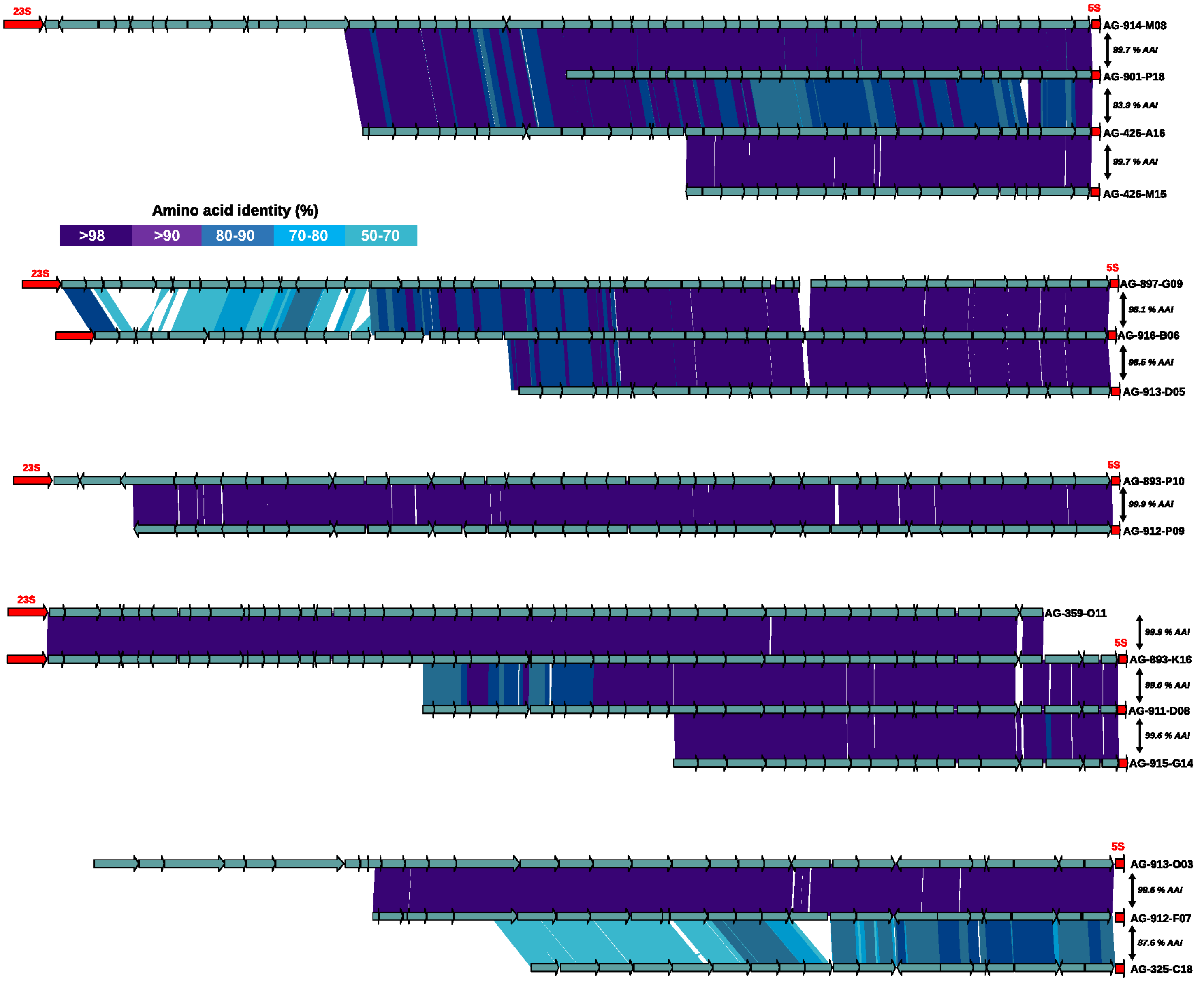

Figure S3. Examples of OBC-type sharing among Pelagibacterales genomes. OBCs are aligned and AAI color-coded.

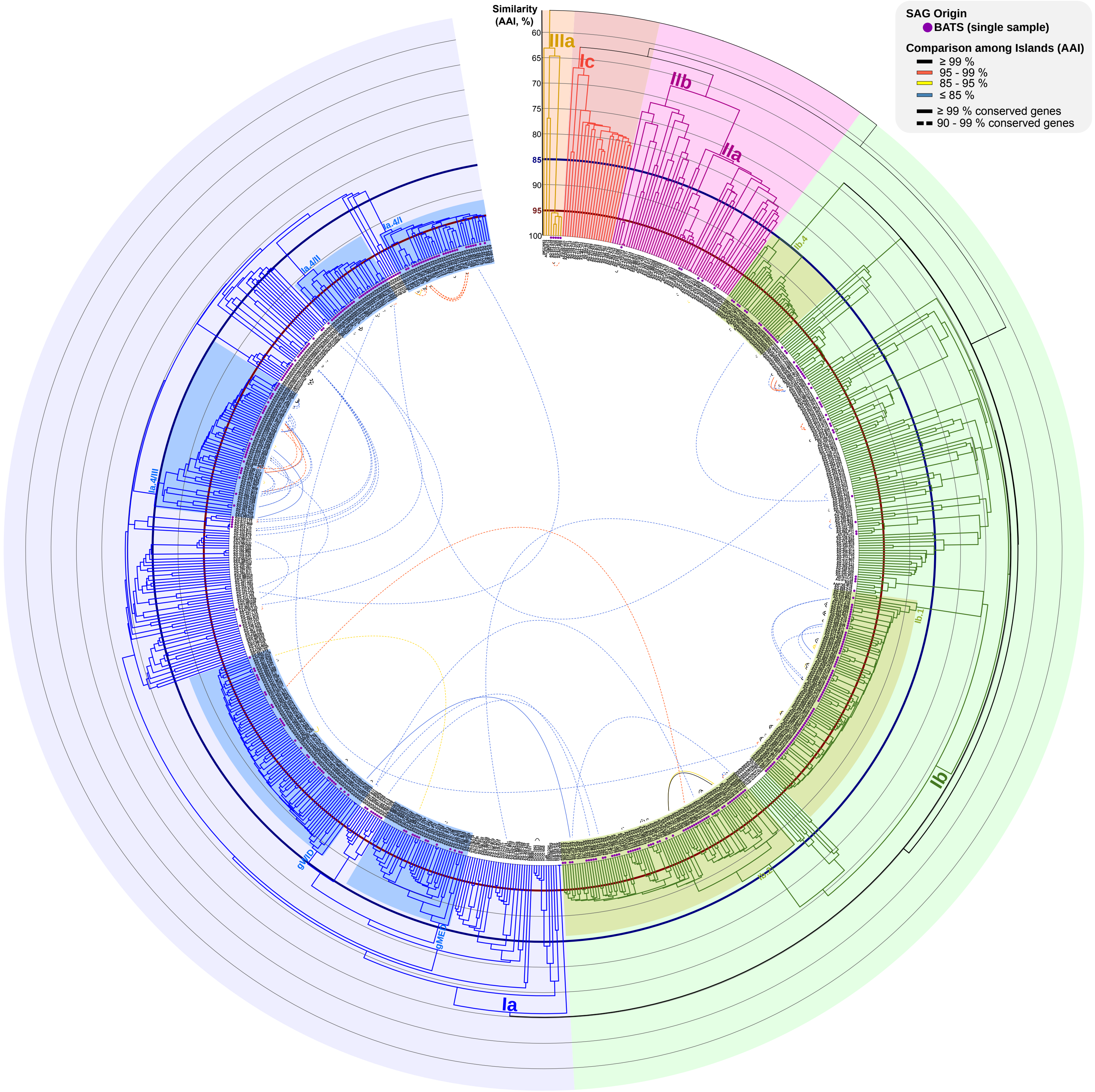

Figure S4. Cladogram-based classification of the 806 genomes containing an OBC, represented as in Figure 1.

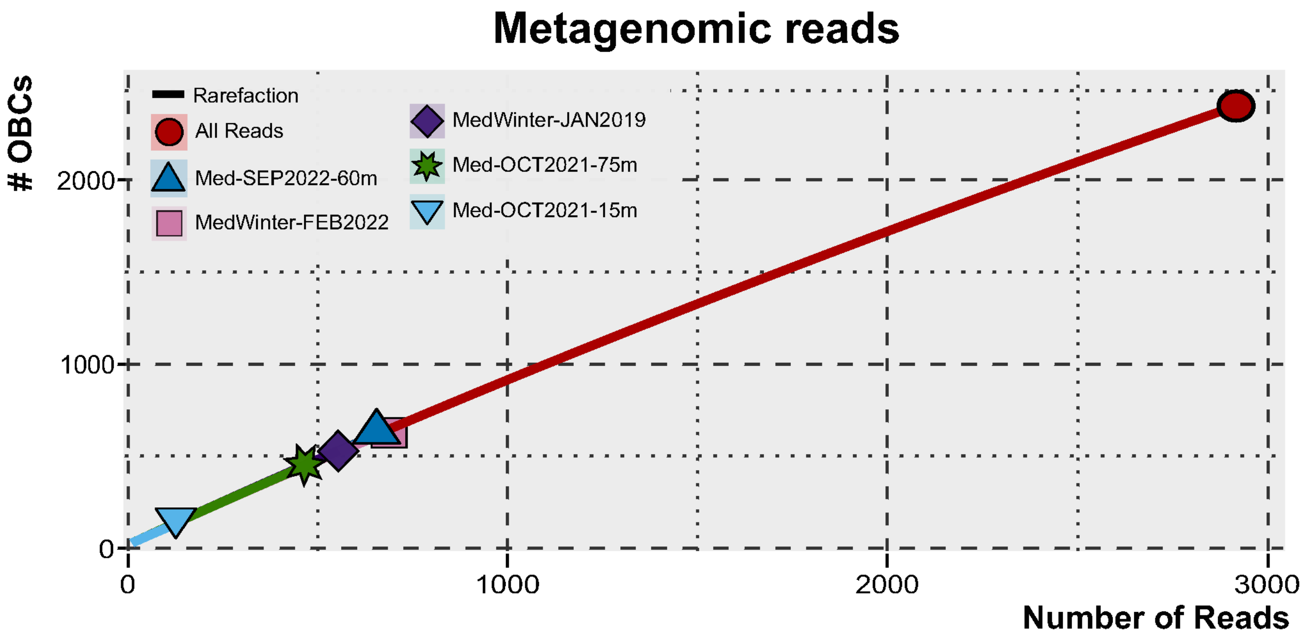

Figure S5. Rarefaction (solid line) curves based on OBC diversity against the number of sequences from a set of five PacBio Sequel II metagenomic reads collected from the Mediterranean Sea.
